# Monitoring and Identification of Sepsis Development through a Composite Measure of Heart Rate Variability

**DOI:** 10.1371/journal.pone.0045666

**Published:** 2012-09-19

**Authors:** Andrea Bravi, Geoffrey Green, André Longtin, Andrew J. E. Seely

**Affiliations:** 1 Department of Cellular and Molecular Medicine, University of Ottawa, Ottawa, Ontario, Canada; 2 Therapeutic Monitoring Systems Inc., Ottawa, Ontario, Canada; 3 Department of Physics, University of Ottawa, Ottawa, Ontario, Canada; 4 Division of Thoracic Surgery, University of Ottawa, Ottawa, Ontario, Canada; 5 Department of Critical Care Medicine, University of Ottawa, Ottawa, Ontario, Canada; University of Adelaide, Australia

## Abstract

Tracking the physiological conditions of a patient developing infection is of utmost importance to provide optimal care at an early stage. This work presents a procedure to integrate multiple measures of heart rate variability into a unique measure for the tracking of sepsis development. An early warning system is used to illustrate its potential clinical value. The study involved 17 adults (age median 51 (interquartile range 46–62)) who experienced a period of neutropenia following chemoradiotherapy and bone marrow transplant; 14 developed sepsis, and 3 did not. A comprehensive panel (N = 92) of variability measures was calculated for 5 min-windows throughout the period of monitoring (12±4 days). Variability measures underwent filtering and two steps of data reduction with the objective of enhancing the information related to the greatest degree of change. The proposed composite measure was capable of tracking the development of sepsis in 12 out of 14 patients. Simulating a real-time monitoring setting, the sum of the energy over the very low frequency range of the composite measure was used to classify the probability of developing sepsis. The composite revealed information about the onset of sepsis about 60 hours (median value) before of sepsis diagnosis. In a real monitoring setting this quicker detection time would be associated to increased efficacy in the treatment of sepsis, therefore highlighting the potential clinical utility of a composite measure of variability.

## Introduction

Tracking the physiological conditions of a patient is of utmost importance in a clinical setting. Treatments provided at an early stage of development of disease are indeed more likely to be effective, and the effectiveness is related to higher chances of survival and lower healthcare costs. Considering the development of severe sepsis, a retrospective study on 2,731 subjects showed that each hour of delay in the initiation of effective antimicrobial therapy is associated with a mean decrease in survival of 7.6% [1]. Furthermore, severe sepsis and septic shock are the most common causes of mortality in critically ill patients, with a mortality of approximately 50% and an average annual cost of $16.7 billion in the USA [2].

Despite the intensive research for the management of severe sepsis and septic shock, there is the lack of a tool capable to continuously monitor its development. In the domain of neonatal sepsis identification, Moorman et al. proposed the Heart Rate Characteristic [3], [4], a logistic regression model combining variability measures applied to R-R interval time. Their approach demonstrated a remarkable reduction in all cause infant mortality in a 3000 patient randomized controlled trial [5], and is leading to commercial applications. However, stepping from neonatal to adult monitoring the scenario changes. Infection remains a clinical diagnosis confirmed in a delayed and insensitive matter by blood cultures, still the gold standard [6], [7]. We first reported on continuous heart rate variability (HRV) measurements during the onset and resolution of clinically diagnosed infection in immuno-compromised ambulatory patients, finding altered HRV in different HRV metrics occurring ∼24–40 hours in advance [8]. In this study we re-analyze the data using a novel method to integrate altered variability.

The present article proposes a novel method, a composite measure of variability, to be used for the identification and tracking of sepsis development. The composite measure was created by applying a sequence of signal processing steps designed to enhance the change from a baseline of health, and integrate the clinically relevant information collected from 92 measures of variability. This broad number of measures was used to maximize the probability to detect clinically useful information from the R-R interval time series. These steps produced a unique, composite measure, which has potential to enrich clinical monitoring.

## Materials and Methods

### Dataset

The study included 21 ambulatory outpatients (age median 51 (interquartile range 46–62)) who underwent bone marrow transplant (BMT) for hematological malignancy or other disorders. Sepsis was defined as systemic inflammatory response syndrome along with clinically suspected infection requiring treatment. Over 50% of the patients had sepsis diagnosed based on the presence of fever, defined a priori as one recording greater than 38.5 degrees centigrade or two recordings greater than 38.0 degrees centigrade within 12 hours. Inclusion criteria were treatment with myeloablative chemoradiotherapy followed by an allogeneic or autologous BMT, and informed consent. Exclusion criteria were pre-existing cardiopulmonary disease, taking beta-blockers or calcium-channel blockers, pre-existing arrhythmia (e.g. atrial fibrillation, atrial bigeminy), contraindication to electrocardiogram adhesives (e.g. allergy, severe psoriasis). Continuous Holter ECG data was collected (average 12 [SD 4] days of monitoring) for all patients in the study, starting approximately 24 h before their BMT and continuing through neutropenia until its resolution or until withdrawal from the study. The used Holter system, a Zymed DigiTrak-Plus (Philips Healthcare, Markham, Ontario, Canada), sampled the ECG at 175 Hz with 10-bit amplitude resolution, and annotated all normal QRS peaks and arrhythmias, including premature atrial and ventricular beats. Only the beats that characterized normal sinus rhythm (NSR) were included, while all premature beats were excluded. RR intervals were derived from R wave annotations. Among the 21 patients, four patients dropped out within 24 h of initiation of monitoring due to discomfort or other reasons, leaving 17 subjects for analysis, 14 of which developed sepsis. Sepsis was defined as systemic inflammatory response syndrome along with clinically suspected infection requiring treatment. Written informed consent was obtained from all participants, and the Ottawa Hospital Research Ethics Board authorized the study. For further details refer to [8].

### Signal Processing

Through a windowed analysis (5 minutes window size, 2.5 minutes overlap) of the RR interval time series, 92 variability time series were extracted for each subject. From now on we will refer to the variability time series with the word “measures”, for simplicity. All the subjects who developed sepsis, developed it after 6 days after admission (median value). Therefore, to reduce the fluctuations with time scales shorter than the time scale of sepsis development, the measures were filtered through a Savitzky-Golay zeroth-order filter (length of 577 samples, i.e. ∼24 hour). Given their considerable number, two data reduction steps were applied: one with the aim of selecting only the relevant information for this specific application, and the other with the aim of reducing the redundant information.

In the first step, the Spearman correlation coefficient (SCC) between the measures and a prototype function representing the expected trend during the development of sepsis was computed. The type of prototype function was arbitrarily chosen as a straight line going from +1 at admission time to −1 to the time of administration of antibiotics. The set of values [−1.+1] is arbitrary, because the SCC is a nonlinear operator which compares only the order between the values of the line and one measure of variability. Those values were specified only to highlight that the correlation between this line and a variability measure is positive only when there is a monotone negative relationship between the two (i.e. the measure is decreasing over time). The values of correlation were then bootstrapped 1000 times to get an estimate of the average population correlation for each measure. Then, 11 measures with the highest average correlation were selected (the number of measures that were selected is arbitrary, and due to keep only ∼10% of the available measures).

In the second step of data reduction, the 11 survived measures (per patient) were processed through Principal Component Analysis (PCA) after admission condition normalization. This normalization transforms the value of the measures into a percentage of change with respect to the first 24 hours after admission, according to the formula ÄHRV = [current – baseline]/range, where “baseline” is the mean variability for the first 24 hours after admission, and “range” is the maximum variability less the minimum variability within the same time frame. PCA is separately applied to each patient, taking the set of 11 measures, and computing the loading coefficients of the first principal component, which is the component oriented in the direction of maximum variance of the dataset [9]. Recalling that the loading coefficients represent the components of a vector, it is possible to create a unique population reference coordinate system by taking the median for each set of loading coefficients across the population. Given the population reference system, the projection of all the measures for each subject is computed, obtaining one time series per subject, which we call a composite measure of variability, or “composite”. To make the model more robust, the population medians of the loading coefficients were computed by bootstrapping 1000 times their distributions.

To motivate the choice of creating a composite measure, for each subject we compared the changes in the composite composite variability with the relative changes (i.e. after admission condition normalization) of the single measures composing it.

To create an alert system identifying when a patient is developing sepsis, the information obtained from the composite measure time series was further reduced by extracting the sum of the energy of the series at very low frequencies, which represent those frequencies with the time scale of sepsis development (i.e. days). This summed energy, which we call *E_s_* for simplicity, was computed in the interval (0,6.3] µHz. This interval was selected to include the peak frequency characterizing each composite measure (see Figure S1). The energy was extracted by computing the Discrete Fourier Transform of the composite and summing the absolute value of the transform in the specified frequential interval.

Approaching the alert system creation from a classification perspective, two classes were introduced; the first one is the *E_s_* during the second day after admission (we remind that the first day was used to pursue the admission condition normalization), namely *E_s1_*, the second one is the *E_s_* from the second day from admission to the moment of administration of antibiotics, namely *E_s2_*. Then, in the training phase a logistic regression was used to create the decision boundary between the two classes, providing the probability of being developing sepsis; only the subjects who developed sepsis were considered in this phase.

**Figure 1:**
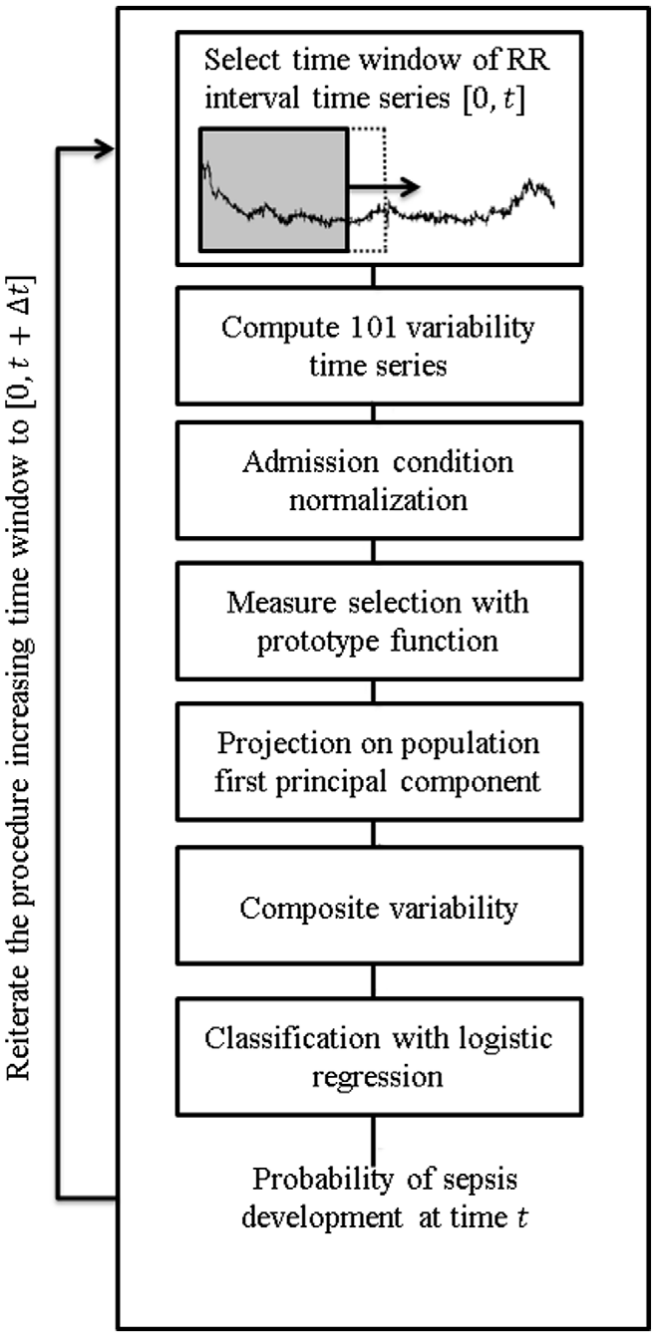
Signal processing diagram. Block diagram showing how to create the composite measure of variability and the likelihood of developing sepsis. The time window [**0**,***t***] is increased at every iteration of 2.5 minutes. This allows to reproduce a monitoring situation where new R-R intervals are continuously analyzed. Having the variability up to at a certain time ***t***, we can compute the composite, and from the composite the probability of developing sepsis.

**Figure 2:**
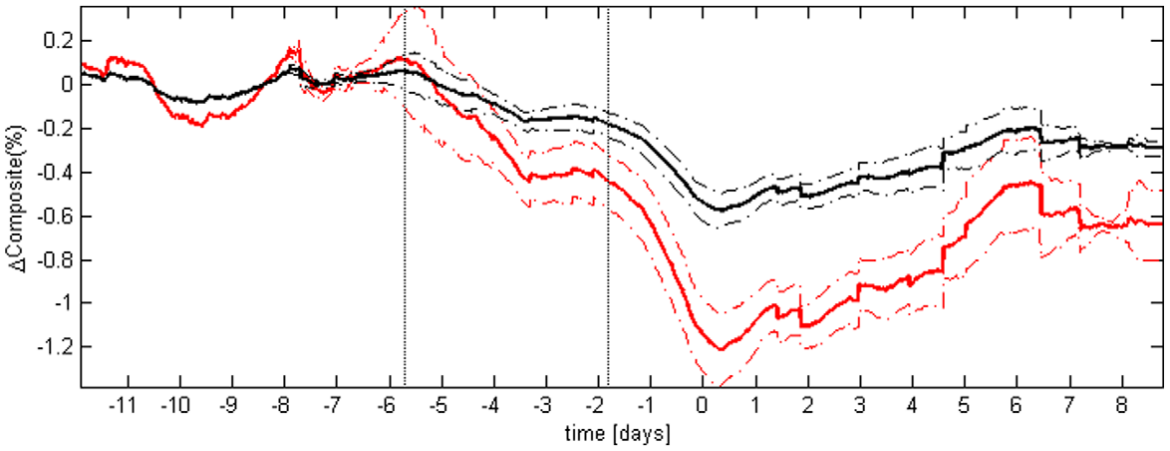
Average composite measure of variability. In red are displayed the results of the composite; for comparison, in black are displayed the results of the detrended fluctuation analysis area under the curve, after admission condition normalization. The continuous lines represent the average value of the time series across the population, and the dashed lines represent plus or minus the standard error of the mean. The two vertical dotted lines highlight when, on average, the composite variability started to drop. Before averaging, for each of the 14 subjects developing sepsis the time series of either the composite or the detrended fluctuation analysis were aligned to the time of administration of antibiotics (t = 0). The picture shows the higher sensitivity of the composite to sepsis development, respect to the sensitivity of a single HRV measure.

Using a leave-one-out cross-validation, we tested the decision boundary for both subjects who developed and did not develop sepsis. To reproduce a standard monitoring situation, the probability of developing sepsis was assessed continuously over time by computing *E_s_* over increasingly long intervals. This means that the same RR interval time series was analyzed taking multiple incremental windows (e.g. the probability is computed by taking the first 30 minutes of data, then the first 60 minutes, then 90, etc…) and reproducing the processing steps described above. To better simulate a monitoring situation, a new value of the probability was computed every 2.5 minutes (i.e. the window step of the windowed analysis). A diagram of the entire processing is reported in Figure 1. Because the *E_s_* is a feature that increases either when variability is decreasing or increasing with frequencies lower than 6.3 µHz, we imposed the probability to be zero when the tested composite measure from which *E_s_* is extracted was increasing or its last value in time was positive (the composite was considered increasing if the SCC with a line going from −1 to +1 was higher than zero). This approach, based on the idea that only a decrease in variability corresponds to physiological impairment, is justified by the fact that the composite measures were found positive and increasing for the subjects who did not develop sepsis (Figure S2), and negative and decreasing when sepsis was developed (Figure 2) (see also Discussion).

## Results

### Composite Measure of Variability

To create a measure tracking the physiological condition of patients undergoing sepsis development, two major signal processing steps are introduced after the computation of 92 variability measures: 1) selection of the informative measures based on nonlinear correlation assessed with a prototype function reflecting decreasing variability, and 2) projection of the selected variability measures onto the first principal component of the population. In the first step, the Spearman’s nonlinear correlation between the time course of the measure and the prototype function is computed for every measure from every subject; then, the average population correlation was computed through bootstrap. The thresholding procedure (see Methods), which aims to retain 10% of the measures with the higher correlation, resulted in 11 surviving variability measures (see Table 1 for details).

**Table 1:** Selected measures of variability.

Measure number	Measure name	Short description
1	Standard deviation	Measure the dispersion of the data from its mean value.
2	Coefficient of variation	Ratio between the standard deviation and the mean of the distribution.
3	Power law Y intercept	After the power spectrum of the time series is computed, a line is fitted in the frequency range [10^−4^], Hz. This measure is the value of the intercept of the y-axis of that line. The power spectrum was computed using the Welch’s method on the interpolated R-R interval, after spline interpolation at 4 Hz.
4	Detrended fluctuation analysisarea under the curve	This measure computes how the variance of the signal change within certain time scales. The area under the curve is the trapezoid integral of the variance-time scales curve.
5	Wavelet area under the curve	Area under the curve of the Wavelet spectral density [14].
6	Shannon entropy	Measure of the degree of complexity of a time series, is based on a weighted sum of the probability of occurrence of a certain.
7	Plotkin-Swamy average energy	The PS energy operator provides a nonlinear estimate of the energy of the signal at a given time. This measure is the average over that energy.
8	Fuzzy entropy	Similarly to sample entropy, fuzzy entropy computes the conditional probability that a pattern seen in an m-dimensional space, could be seen in a (m+1)-dimensional space. The difference is that, to assess whether two points in the phase space are close, a fuzzy membership function is used instead of a Heaviside step function.
9	Correlation dimension Global	Measure of the dimensionality of a time series attractor.
10	Cardiac vagal index	This measure is a combination of the two orthogonal spreads in the Poincaré plot.
11	Largest Lyapunov exponent	Measure quantifying the chaoticity of a system.

For further details about these measures refer to [10]. The specific parameters used to compute the measures are available upon request.

The survived techniques were projected onto the population first principal component, which on average accounted for 95.6% of the variance of the measures. Those techniques found different relevance inside the first principal component, as showed in Figure S3. Looking at the median of the loading coefficients across the population, the area under the curve of the detrended fluctuation analysis (selected measure number 4), the wavelet area under the curve (measure number 5) and the Shannon entropy (measure number 6) were identified as having the highest values. However, no real major contributor to the principal component was found, because all the techniques had close loading coefficients.

The composite measure of variability created through this process presented a clear decline over time for patients who developed sepsis (see Figure 2). The decline started on average around 140 hours prior the administration of antibiotics, increasing its speed at about 42 hours prior. For comparison we also show the composite for the three subjects who did not develop sepsis in Figure S2. The composite in that case showed positive values, and was either increasing or oscillating around positive values. The comparison of the composite variability with its single components showed that the projection on the principal component produced changes 2 to 3 times larger than any single measure. For simplicity we reported only the change of the detrendended fluctuation analysis area under the curve (DFA AUC, i.e. measure #4), being the measure with a higher weight in the PCA model, and therefore a major contributor to the composite (see Figure 2).

### Detection of Sepsis Development

**Figure 3:**
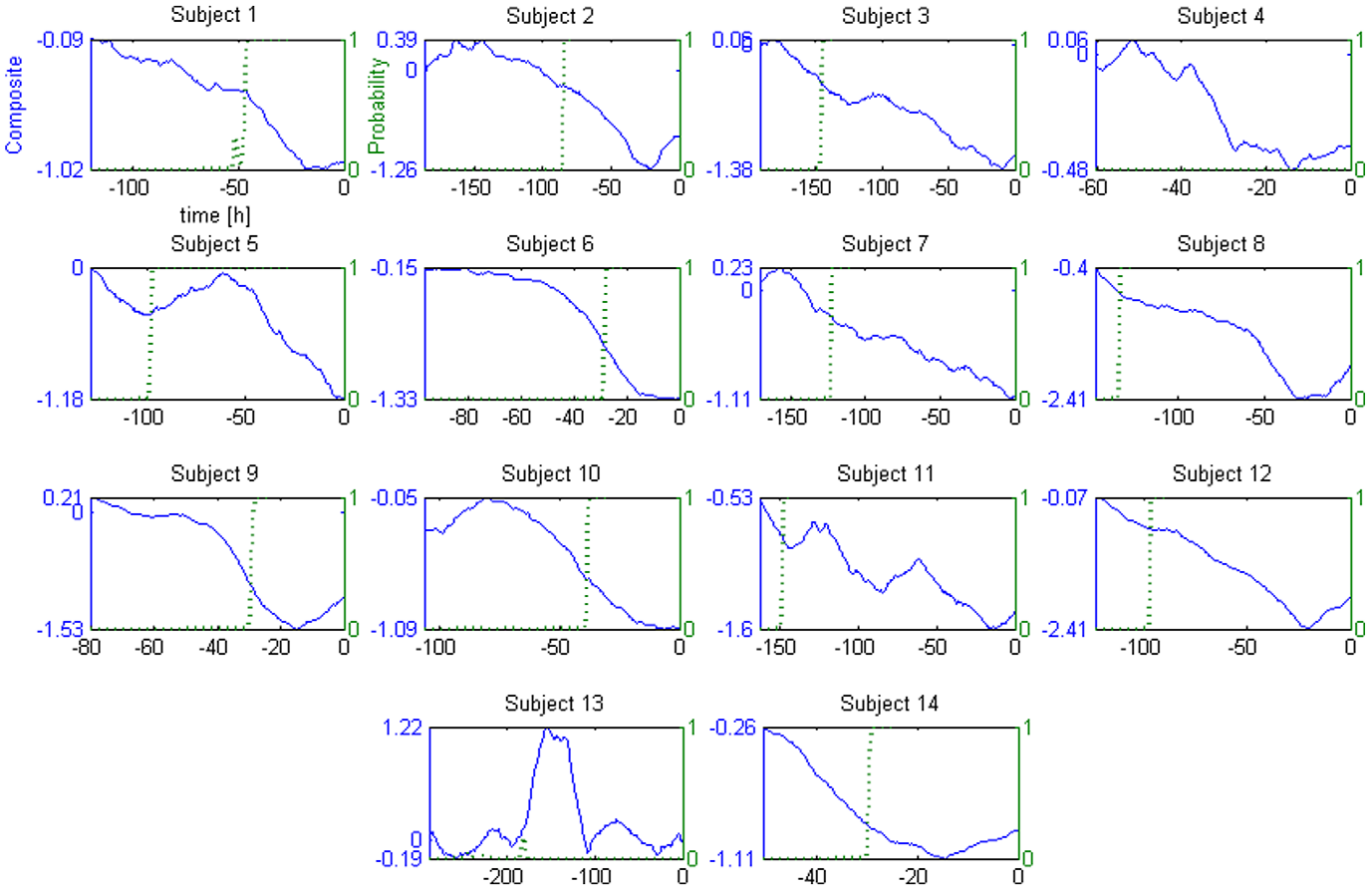
Probability of development of sepsis. This set of double graphs show the composite measure of variability (blue solid line) and the probability of developing sepsis (green dotted line) at a given time, for each subject. As reported for the plot of subject 1, the x-axis is the time with respect to the administration of antibiotics (t = 0).

To detect sepsis development the summed energy at the very low frequencies (*E_s_*) was extracted from the composite. This feature represents the energy at the frequencies with the same time scale of sepsis development. The detection of sepsis development was achieved by training a logistic regression with two classes of data, one representing early health conditions (*E_s_* of the second day after admission, called *E_s1_*), the other representing sepsis at its most advanced stage (*E_s_* from second day to administration of antibiotics, called *E_s2_*). The two classes show considerably different values (Figure S4), making the classification task easier. We also computed the energy at the very low frequency for the single measures of variability composing the composite, after admission condition normalization. All of them showed a considerably lower separation between the two classes. Nevertheless, in all cases (composite and single measures) the differences between *E_s1_* and *E_s2_* allowed rapid detection of the development of sepsis when tracking over time the probability estimated from the logistic regression (Figure 3). This was true for all the subjects except subjects 4 and 13; this result is discussed below. The median time of detection of sepsis development was 60 hours in advance (the values for each subject are reported in Table 2). Sepsis development was considered “detected” when the probability reached values higher than 0.99. The transition time from probability zero to detection had a median value of 3.3 hours. The early warning system was run also on the three subjects who did not develop sepsis; as expectable, the estimated probability of developing sepsis remained stable at zero for all of them. The classification performances of the system were: sensitivity 86% (12/14), specificity 100% (3/3), positive predictive value 100% (12/12), and negative predictive value 60% (3/5).

**Table 2:** Detection of sepsis development through composite variability.

Subject number	Detection time[hours in advance]	Transition time[hours]
1	45.42	7.91
2	84.17	0.41
3	144.2	2.9
4	0	–
5	96.25	3.33
6	27.25	2.33
7	121.7	2.1
8	133.3	1.7
9	27.92	2.5
10	37.5	2.08
11	147.5	2.5
12	96.25	1.25
13	0	–
14	29.17	1.25

Median value of detection 64.7 hours in advance, with a transition time from probability zero to probability higher than 0.99 of 3.3 hours (median value).

## Discussion

In this paper a procedure to create a composite measure tracking the change in variability during the development of sepsis was presented. Being based on a dataset of ambulatory outpatients, the procedure makes the major assumption that a patient started being monitored before he actually developed sepsis, or at an early stage of sepsis development. This assumption limits the applicability of the procedure to only a patient population which starts from a “baseline of health”. However, this specificity is expected to provide improved outcome because taking advantage of a piece of information, i.e. the drop in variability, which would not be present if the patient was already in critical conditions, maybe because of multiple organ dysfunction syndrome (therefore presenting already a low variability).

The creation of the composite measure targeted to sepsis development required two major steps: 1) a selection based on nonlinear correlation with a prototype function representing decreasing variability, which we hypothesize is related to altered clinical physiological state; and 2) the projection on the first principal component of the population of the selected measures, which magnified the information relative to the change in variability. Comparing the trends of the composite measure for both subjects who did (Figure 2) and did not develop sepsis (Figure S2) a distinction appeared, both from the point of view of the trends (the former decreasing, the latter increasing or stable), and of the values of the measure (the former negative, the latter positive). Our system was able to properly identify 15 subjects out of 17, both developing and not developing sepsis. As discussed previously, these results are supported by a growing literature documenting a reduction in HRV correlates with infection and its severity [11]. Other recent examples include the use of short-term heart and respiratory rate variability, combined with other physiological parameters, to predict the risk of severe morbidity due to infection on 138 preterm infants [12], and the use of HRV to predict post-stroke infection [13].

To motivate the creation of the composite measure, for each subject we compared the relative change (i.e. after admission condition normalization) in the composite with the relative changes of the single measures composing it. We found the composites showed larger changes respect to the single measures. This is not surprising given that the first principal component is a weighted sum of those single changes. The result was confirmed also by the comparison of the sum of the energy at the very low frequencies. This makes the principal component analysis a preferred tool to create a composite measure of variability for this specific application.

To further illustrate the potential clinical utility of the composite measure, we also proposed an early warning system based on the sum of the energy of the composite. We selected the sum of the energy of the composite as the feature of interest after the extraction of a variety of other features, which however did not provide the same results in terms of quality of the prediction of sepsis (see next). Indeed, the chosen system provided in the majority of the cases smooth probability transitions, detecting several hours in advance the development of sepsis. An exception was subject 4, the one with the lowest *E_s_* (Figure S4). Because his *E_s_* was not presented to the logistic regression at the time of creation of the model (we used the leave-one-out cross validation), the decision boundary of the classifier resulted in a value higher than his *E_s_*; this produced a probability of zero. Therefore, this false negative represents an effect of the small sample size. The second exception is subject 13, and is due to the fact that his composite measure had for the majority of the recording positive, and increasing values (Figure 3). In this case a drop in variability did not correspond to physiological deterioration, rather the opposite. Further investigations are needed to explain why this subject expressed a different trend in HRV. Nevertheless, the present study shows how the hypothesis of direct proportionality between variability and health might be usefully employed for individual patients.

The composite measure showed improved results (i.e. faster detection time and shorter transition time) respect to the measures with a lower weight in the PCA, and results comparable to the ones of the measures with a large weight in the PCA (such as DFA AUC). This is related to the fact that the observed changes in HRV associated to sepsis resulted large enough not to require the increased sensitivity of the composite (i.e. there was a significant separation between the distributions of Figure S4). Nevertheless, this sensitivity would prove useful in detecting sepsis development when more subtle changes in HRV appeared. For instance subjects developing sepsis faster than what we observed with this dataset would present a smaller energy at the very low frequencies, therefore making the increased sensitivity of the composite useful in better detecting sepsis development.

There are several limitations to this study. While its purpose is principally to introduce a method to integrate numerous variability metrics into a single composite measure, the clinical evaluation is limited to a small pilot dataset. Furthermore, during the design we made a few arbitrary choices which require validation. Some were justified by intuition, such as the choice of the frequency range to compute the *E_s_*, identified by inspection of Figure S1. Others were justified based on results across multiple trials. For example, we selected different filter lengths, types of normalization and number of variability measures to include in the principal component model to improve the smoothness of the profiles of the composite measure and of the probability of developing sepsis. A wider analysis that better motivates these choices would be beneficial not only in further improving the performance, but also in understanding the independent value of each measure of variability. Furthermore, as mentioned, the proposed results are based on a pilot study, involving only few patients. Thus, the usefulness of the proposed approach needs to be proven on larger datasets.

In summary, the composite measure here proposed represents a tool which joins the clinically valuable information of several measures of variability, in a specific way targeted to enhance the sensitivity to sepsis development. This approach addresses a key challenge in variability analysis, which is the reduction of the dimensionality of the analysis, given by the large number of measures currently available [10]. The idea of merging the information from different measures through data reduction techniques could be applied in future studies to merge information streams from multiple organs. This study, for the first time, presents a methodology with which to combine multiple variability metrics in a feasible and potentially clinically useful manner.

## Supporting Information

Figure S1 **Fourier transform of the composite.** The figure shows the Fourier transform of the composite measure of variability for each subject. The black vertical line represents the threshold of 6.3 µHz, which was arbitrarly chosen to include the majority of the energy of the signals (i.e. the peaks in the transform). The energy at the very low frequency *E_s_* was defined as the sum of the energy in the frequency interval (0, 6.3] µHz. A different selection of the threshold did not produce any change in the results, as long as those peaks were included.
(TIF)

Figure S2 **Composite of subjects who did not develop sepsis.** These time series represent the composite measures computed for the three subjects who did not develop sepsis. Because the admission condition normalization takes out the first day of recording, the composite starts from 24 hours after admission. The constant value at the end of the composite is due to the transient of the Savitzky-Golay filter.
(TIF)

Figure S3 **Population PCA.** These panels summarize the model built from the PCA. The list of the eleven variability measures employed in the model, together with their relative number and short description, is reported in Table 1. The upper panel above shows the values of the loading coefficients of the first component for each of the 14 subjects who developed sepsis, and each of the 11 measures which survived the nonlinear correlation selection. Each measure contributed slightly differently to the PCA, depending on the patient. The lower panel represents the same data in the form of median and interquartile error after bootstrapping 1000 times the loading coefficients distributions. Please note that the number identifying the subjects in the figure above (y-axis) do not correspond to the subject numbers reported in [**8**]; indeed this numbering excludes the subjects who did not develop sepsis.
(TIF)

Figure S4 **Energy of the composite.** The bars represent the energy at the very low frequencies (*E_s_*) of the composite measures for all the subjects during the second day, i.e. *E_s1_*, and from the second day to antibiotics administration, i.e. *E_s2_*. In colors are the energies extracted from the composite measure of variability, in black are over-imposed the energies extracted from the detrended fluctuation analysis area under the curve after admission condition normalization. The composite showed a larger separation between the two classes, and therefore increased sensitivity to sepsis development. A Wilcoxon signed-rank test showed that the null hypothesis of equal median between *E_s1_* and *E_s2_* is rejected, for both the composite and the single measure of variability (p-value ∼10^−4^).
(TIF)
