# Supplementary figures and images for: Monitoring and Identification of Sepsis Development through a Composite Measure of Heart Rate Variability
Source: PLoS One. 2012 Sep 19;7(9):e45666. doi: 10.1371/journal.pone.0045666 (PMC3446945; doi:10.1371/journal.pone.0045666)

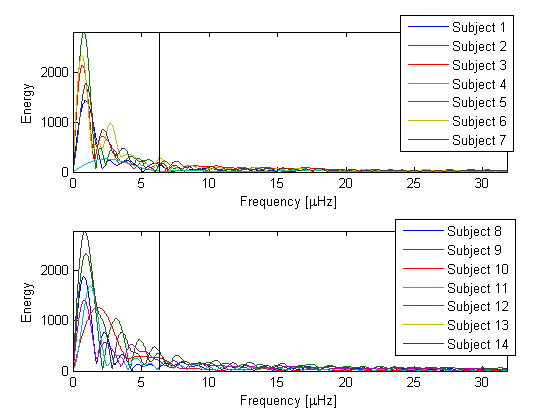

Supplement: Figure S1 — Fourier transform of the composite. The figure shows the Fourier transform of the composite measure of variability for each subject. The black vertical line represents the threshold of 6.3 µHz, which was arbitrarly chosen to include the majority of the energy of the signals (i.e. the peaks in the transform). The energy at the very low frequency Es was defined as the sum of the energy in the frequency interval (0, 6.3] µHz. A different selection of the threshold did not produce any change in the results, as long as those peaks were included. (TIF) [file pone.0045666.s001.tif]

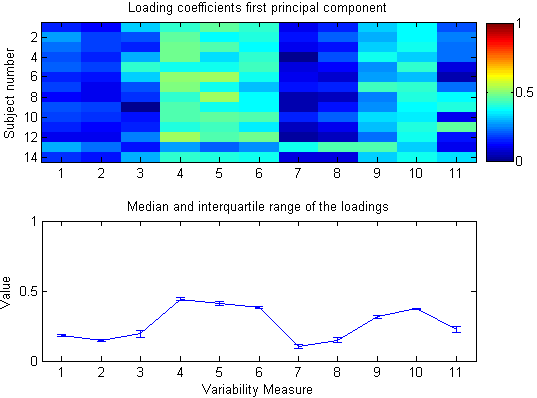

Supplement: Figure S3 — Population PCA. These panels summarize the model built from the PCA. The list of the eleven variability measures employed in the model, together with their relative number and short description, is reported in Table 1. The upper panel above shows the values of the loading coefficients of the first component for each of the 14 subjects who developed sepsis, and each of the 11 measures which survived the nonlinear correlation selection. Each measure contributed slightly differently to the PCA, depending on the patient. The lower panel represents the same data in the form of median and interquartile error after bootstrapping 1000 times the loading coefficients distributions. Please note that the number identifying the subjects in the figure above (y-axis) do not correspond to the subject numbers reported in [8]; indeed this numbering excludes the subjects who did not develop sepsis. (TIF) [file pone.0045666.s003.tif]

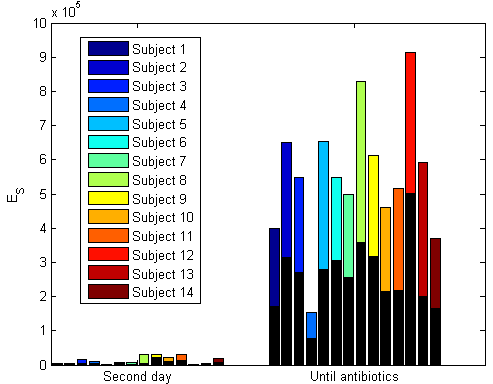

Supplement: Figure S4 — Energy of the composite. The bars represent the energy at the very low frequencies (Es) of the composite measures for all the subjects during the second day, i.e. Es1, and from the second day to antibiotics administration, i.e. Es2. In colors are the energies extracted from the composite measure of variability, in black are over-imposed the energies extracted from the detrended fluctuation analysis area under the curve after admission condition normalization. The composite showed a larger separation between the two classes, and therefore increased sensitivity to sepsis development. A Wilcoxon signed-rank test showed that the null hypothesis of equal median between Es1 and Es2 is rejected, for both the composite and the single measure of variability (p-value ∼10−4). (TIF) [file pone.0045666.s004.tif]
